# An intercalated BSc degree is associated with higher marks in subsequent medical school examinations

**DOI:** 10.1186/1472-6920-9-24

**Published:** 2009-05-19

**Authors:** Jennifer A Cleland, Andrew Milne, Hazel Sinclair, Amanda J Lee

**Affiliations:** 1 School of Medicine and Dentistry, University of Aberdeen, Foresterhill Health Centre, Westburn Road, Aberdeen, UK

## Abstract

**Background:**

To compare medical students on a modern MBChB programme who did an optional intercalated degree with their peers who did not intercalate; in particular, to monitor performance in subsequent undergraduate degree exams.

**Methods:**

This was a retrospective, observational study of anonymised databases of medical student assessment outcomes. Data were accessed for graduates, University of Aberdeen Medical School, Scotland, UK, from the years 2003 to 2007 (n = 861). The main outcome measure was marks for summative degree assessments taken after intercalating.

**Results:**

Of 861 medical students, 154 (17.9%) students did an intercalated degree. After adjustment for cohort, maturity, gender and baseline (3^rd ^year) performance in matching exam type, having done an IC degree was significantly associated with attaining high (18–20) common assessment scale (CAS) marks in three of the six degree assessments occurring after the IC students rejoined the course: the 4^th ^year written exam (p < 0.001), 4^th ^year OSCE (p = 0.001) and the 5^th ^year Elective project (p = 0.010).

**Conclusion:**

Intercalating was associated with improved performance in Years 4 and 5 of the MBChB. This improved performance will further contribute to higher academic ranking for Foundation Year posts. Long-term follow-up is required to identify if doing an optional intercalated degree as part of a modern medical degree is associated with following a career in academic medicine.

## Background

Traditionally, about one-third of UK medical students undertake a year additional to the basic five year undergraduate course to intercalate a degree. However, this figure varies widely across medical schools (with the exception of graduate entry courses where students already have equivalent qualifications), from 5% to 100% [1].

There appear to be a number of benefits to doing an intercalated degree. A longitudinal study of all UK medical school graduates in 1996 and 1997 found students who had taken an intercalated degree had higher strategic and deep learning scores and lower surface learning scores than those who had not [2]. Intercalating is frequently suggested as one method of planning for a career in academic medicine [3] and there is evidence that doing an intercalated degree encourages entry into academic careers [4,5] and, consequently, those with intercalated degrees are more likely to have papers published in refereed scientific journals [4,6] and attract research grants [6]. There are also a number of suggested, but unsubstantiated, benefits such as having increased ability to critically evaluate research and understand methodological principles [7].

However, other factors seem to discourage medical students from intercalating. One potential factor is the norm of the medical school: if these degrees are perceived to be only for high achievers, then many students will not consider this option [7]. Where such degrees are obligatory, students accept this commitment when they apply for medical school. Another might be lack of exposure to academia, and thus little awareness of this as a potential career option; indeed few doctors choose academic medicine as a career [7-9]. Clearly, other significant barriers to intercalating perceived by students are time and financial costs.

UK Medical training has changed in recent years. The introduction of Modernising Medical Careers [10] encourages young doctors to make decisions about their future career pathways at a much earlier stage of training. Intercalating (or having another degree) gained additional points in the academic ranking system on the Medical Training and Application Service (MTAS) for matching candidates to the Foundation Year (FY) Programme posts. Although MTAS was abandoned in June 2007, ranking will continue to be used in any new matching system ( 2nd August 2007). Ranking also depends on the average of the student's performance over the medical degree: better performance leads to higher ranking which is beneficial when applying for competitive posts. Thus any short-term benefit to intercalating, in terms of improved performance in subsequent undergraduate degree exams (over and above the additional points for attaining another degree), may attract students to doing an intercalated degree

Unfortunately, those studies which have examined intercalating student performance on rejoining the medical course provide conflicting evidence [11,12], involve low numbers of students [12], selected students (only those intercalating pathology [11]) and are, with respect to the authors, now dated given the changes to the structure and content (the introduction of Tomorrow's Doctors [13]) of UK medical degrees. While McManus and colleagues [2] identified that students who intercalated gained more effective learning strategies than their non-intercalating peers, they did not compare performance on degree assessments across groups. Thus, the immediate gains from intercalating are unclear.

This study aimed to compare medical students on a modern MBChB programme who did an optional intercalated degree with their peers who did not intercalate; in particular, to monitor performance in subsequent undergraduate degree exams.

The context of this study was a long-established medical school with a distinguished research history. The school is relatively small (annual intake of just under 200 students). At the time of data collection, the medical degree was a 5-year programme with a mostly undergraduate intake. The programme emphasised early clinical experience; had a vertical communication theme; was problem-oriented, with a lecture- and Student Selected Component-based approach to teaching and learning. Comparison of career choices by medical school in graduates of 1999 and 2000 indicated that Graduates from the school under study were more likely to choose General Practice, Anaesthetics, A&E and other medical specialties, and less likely to choose surgical specialties, as their first choice of long-term career than the national average [14].

## Methods

The study subjects were all University of Aberdeen MBChB students who graduated in the years 2003 to 2007. At the time of data collection, the Aberdeen MBChB intercalated degree programme was placed between 3^rd ^and 4^th ^year of the five year basic programme. Students applied mid-3^rd ^year, then must have attained a minimum grade in 3^rd ^year (roughly equivalent to a second class upper degree standard) to be accepted to intercalate. All intercalating (IC) students complete a core course on foundations of medical research, a second set of core topics that are related to clinical or laboratory-based research (depending on the student's research project), then a 20-week research project which is written up as a thesis.

Data on gender, age, funding status (home or overseas), any previous degree and marks are routinely collected during the selection and degree assessment processes.

### Assessment on the MBChB

Marks are collected in the form of the common assessment scale (CAS), a 21-point non-linear scale from 0–20, used for all assessments at the University of Aberdeen.

Only marks for summative degree assessments were examined in this study (see Table 1). These take five forms: Extended Marching Questions (EMQs) [15], Multiple-Choice Questions (MCQs, MEQs) [16], Objective Structured Clinical Exams (OSCEs) [17] and journal-style essay projects (see Table 1). Assessment marks based on group work (either 100% or a significant proportion of the grade; applicable to four summative degree assessments only) were not included as we wished to examine individual student performance. Nor were global ratings of student performance on clinical attachments included as studies indicate that these can often be inconsistent with performance [18,19].

**Table 1:** Details of each assessment entered into the analysis

		**Code**
**Written Degree Exams (Years 1–5)**	**Year 1** Systems I (MCQ) Basic Science for Medicine (MCQ) Systems II (MCQ)	**WR1:1** **WR1:2** **WR1:3**
	**Year 2**	
	Principles of Medicine I (EMQ)	**WR2:1**
	Principles of Medicine II (EMQ and MEQ)	**WR2:2**
	Community Course II (MEQ)	**WR2:3**
	Principles of Medicine III (EMQ and MEQ)	**WR2:4**
	**Year 3**	
	Principles of Medicine IV a (EMQ and MEQ)	**WR3:1**
	Principles of Medicine IV b (EMQ and MEQ)	**WR3:2**
	**Year 4**	
	Specialist Clinical Practice II (EMQ, MEQ, MCQ)	**WR4**
**Clinical (OSCE) exams (Years 3–5)**	**Year 3**	
	Clinical Skills II	**OSCE3**
	**Year 4**	
	Clinical Practice II	**OSCE4**
	**Year 5**	
	Finals	**OSCE5**
**Journal-style paper assessments (Years 3–5)**	**Year 3**	
	Community Course III	**JP3**
	**Year 4**	
	Student Selected Module (SSM)	**JP4**
	**Year 5**	
	Paramedical Elective	**JP5:1**
	Medical Elective	**JP5:2**

SPSS for Windows, Version 16.0 was used for data storage and analysis. The Mann-Whitney test was used to compare the distribution of age across intercalated groups since it followed a non-Normal distribution. Categorical data were described as percentages and associations between intercalation and CAS band were compared using the chi-square test. The Aberdeen CAS system is such that mean scores were not available and therefore effect sizes could not be estimated. Multinomial logistic regression was used to explore the association of intercalating with student performance in the fourth and fifth year assessments. The regression procedure shows the odds of attaining a particular CAS mark band in a specific exam for intercalating versus non-intercalating students. Next, to account for the potential confounding of those who chose to take an intercalated degree after third year being better and/or more mature students from the outset, the following covariates were simultaneously included in each model: baseline (3^rd ^year) performance in matching exam type; cohort; maturity and gender. Note that previous degree was not included as a covariate due to problems of collinearity with the variable 'maturity' since the vast majority of the mature students had a previous degree. The Nagelkerke pseudo R^2^, was also documented. This can take a value of between 0 and 1 and is a marker of the improvement in goodness of fit of the current model over a model containing just the intercept term. If the independent variables in a model perfectly predicted the outcome, then the Nagelkerke pseudo R^2 ^would equal 1. Due to the relatively high number of statistical tests performed throughout the manuscript, and the resulting increase in likelihood of a type 1 error (false positive), a more stringent value of p ≤ 0.01 was used to denote statistical significance throughout all analyses.

Ethical Approval was not required as this was a retrospective analysis of an anonymised database.

## Results

### Characteristics of students

The study included 861 medical students, 178 in the 2003 graduating cohort, 155 in 2004, 177 in 2005, 168 in 2006, and 183 in the 2007 cohort. Overall, the majority were female (442; 51.3%); there were 59 (7%) overseas-funded students and 92 (11%) graduates. One hundred and fifty-four (17.9%) students did an intercalated degree, a median of 31 students per year (range 29–34).

Table 2 provides comparison data for non-intercalating (non-IC; 707) and intercalating (IC; n = 154) students. While not evident by the median or inter-quartile range (IQR), the overall distribution of age indicated that IC students had a significantly younger age at entry than non-IC students (a range of 3 years compared to 16 years respectively). Among those doing an intercalated degree, marginally more of them were female students (78, 50.6%), but this difference was not statistically significant. No overseas-funded students or graduate students did an IC degree.

**Table 2:** Demographic Patterns in Intercalating and Non-Intercalating Students

		**Intercalated** **% (n)**	**Non-Intercalated** **% (n)**	**P-value**^1^
**Age**		18 (17,19)	18 (18,19)	**≤ 0.001**
**Maturity**	School Leaver (< 21 years old)	100 (154)	84 (594)	**≤ 0.001**
	Mature Student (> 21)	0 (0)	16 (113)	
**Gender**	Male	49.4 (76)	48.5 (343)	0.46
	Female	50.6 (78)	51.5 (364)	
**Funding Source**	Home	100.0 (154)	91.7 (648)	**≤ 0.001**
	Overseas	0.0 (0)	8.3 (59)	
**Graduates**	No previous degree	100.0 (154)	87.0 (615)	**≤ 0.001**
	Graduate	0.0 (0)	13.0 (92)	

Figures are % (n) except for age (median (interquartile range))

^1 ^P-value from chi-square test except age which is from Mann Whitney test

Tables 3 and 4 describe differences between IC and non-IC student performance on each individual assessment. Resit data were not included in the analysis, only student performance on the first exam sitting. For presentation purposes, marks were categorised into their locally-used 5 bands: CAS mark 18–20 outstanding = 1; 15–17 very good = 2; 12–14 good = 3; 9–11 pass = 4 and 0–8 = Fail. In Table 3a, note that lower numbers are reported for Basic Science for Medicine as this exam was first run in 1999; thus, two cohorts of students did not sit this exam. Systems I (WR1:1) and Systems II (WR1:3) have slightly lower numbers (825 and 829 respectively) as students who entered directly into 2^nd ^year did not sit these exams.

**Table 3:** Univariate Analysis of Intercalated Student Performance in years 1–3

**Assessment Type**	**Year**	**Exam**	**Mark Band**^1^	**Intercalated** **% (n)**	**Non-Intercalated** **% (n)**	**P-value**^2^
**Written**	**1**	WR1:1 Systems I (n = 825)	1	26.0 (40)	19.2 (129)	0.011
			2	29.2 (45)	25.3 (170)	
			3	29.9 (46)	28.6 (192)	
			4	9.7 (15)	11.9 (80)	
			Fail	5.2 (8)	14.9 (100)	
		WR1:2 Basic Science for Medicine (n = 648)	1	25.8 (31)	11.0 (58)	**< 0.001**
			2	26.7 (32)	21.0 (111)	
			3	25.8 (31)	28.2 (149)	
			4	10.8 (13)	13.8 (73)	
			Fail	10.8 (13)	25.9 (137)	
		WR1:3 Systems II (n = 826)	1	22.1 (34)	12.6 (85)	**0.009**
			2	30.5 (47)	25.3 (170)	
			3	24.7 (38)	31.7 (213)	
			4	11.7 (18)	15.3 (103)	
			Fail	11.0 (17)	15.0 (101)	
	**2**	WR2:1 Principles of Medicine I (n = 861)	1	17.5 (27)	11.5 (81)	0.016
			2	46.1 (71)	39.7 (281)	
			3	29.9 (46)	37.1(262)	
			4	5.8 (9)	11.6 (82)	
			Fail	0.6 (1)	0.1(1)	
		WR2:2 Principles of Medicine II (n = 861)	1	18.2 (28)	11.7 (83)	0.028
			2	47.4 (73)	44.0 (311)	
			3	27.9 (43)	30.8 (218)	
			4	6.5 (10)	11.3 (80)	
			Fail	0 (0)	2.1 (15)	
		WR2:3 Community Course II (n = 861)	1	5.8 (9)	8.5 (60)	0.539
			2	39.0 (60)	33.0 (233)	
			3	37.7(58)	38.5 (272)	
			4	16.2 (25)	18.0 (127)	
			Fail	1.3 (2)	2.1 (15)	
		WE2:4 Principles of Medicine III (n = 861)	1	12.3 (19)	6.9 (49)	**< 0.001**
			2	46.1 (71)	31.1 (220)	
			3	30.5 (47)	30.6 (216)	
			4	10.4 (16)	27.2 (192)	
			Fail	0.6 (1)	4.2 (30)	
	**3**	WR3:1 Principles of Medicine IVa (n = 861)	1	5.8 (9)	6.4 (45)	0.034
			2	20.1 (31)	21.4 (151)	
			3	46.8 (72)	41.7 (295)	
			4	27.3 (42)	24.6 (174)	
			Fail	0 (0)	5.9 (42)	
		WR3:2 Principles of Medicine IVb (n = 861)	1	12.3 (19)	8.1 (57)	**< 0.001**
			2	42.2 (65)	29.1 (206)	
			3	34.4(53)	40.9 (289)	
			4	11.0 (17)	17.7 (125)	
			Fail	0 (0)	4.2 (30)	
**OSCE**	**3**	OSCE3 Clinical Skills (n = 861)	1	12.3 (19)	12.2 (86)	**0.003**
			2	31.8 (49)	34.9 (247)	
			3	46.8 (72)	32.7 (231)	
			4	7.1 (11)	15.4 (109)	
			Fail	1.9 (3)	4.8 (34)	
**Journal-style paper (essay)**	**3**	JP3 Community Course III (n = 861)	1	16.2 (25)	7.6 (54)	**0.003**
			2	52.6 (81)	49.9 (353)	
			3	26.0 (40)	31.1 (220)	
			4	4.5 (7)	10.3 (73)	
			Fail	0.6 (1)	1.0 (7)	

^1 ^Mark bands were categorised into their 5 locally-used bands: 1 = outstanding, CAS marks 18–20; 2 = very good, CAS marks 15–17; 3 = good, CAS marks 12–14; 4 = pass, CAS marks 9–11; Fail = CAS marks 0–8.

^2 ^P-value from chi-square test

**Table 4:** Univariate Analysis of Intercalated Student Performance in years 4–5

**Assessment Type**	**Year**	**Exam**	**Mark Band**^1^	**Intercalated** **% (n)**	**Non-Intercalated** **% (n)**	**P-value**^2^
**Written**	**4**	WR4 Specialist Clinical Practice II (n = 861)	1	14.9 (23)	5.5 (39)	**< 0.001**
			2	50.0 (77)	37.3 (264)	
			3	27.3 (42)	40.0 (283)	
			4	7.1 (11)	15.6 (110)	
			Fail	0.6 (1)	1.6 (11)	
**OSCE**	**4**	OSCE4 Clinical Practice (n = 861)	1	13.0 (20)	7.4 (52)	0.015
			2	43.5 (67)	35.9 (254)	
			3	34.4 (53)	41.4 (293)	
			4	8.4 (13)	12.6 (89)	
			Fail	0.6 (1)	2.7 (19)	
	**5**	OSCE5 Finals (n = 861)	1	20.1 (31)	10.7 (76)	0.025
			2	47.4 (73)	48.8 (345)	
			3	25.3 (39)	31.3 (221)	
			4	5.2 (8)	6.9 (49)	
			Fail	1.9 (3)	2.3 (16)	
**Journal-style paper (essay)**	**4**	JP4 Student Selected Module IV (n = 861)	1	17.5 (27)	13.0 (92)	0.417
			2	63.0 (97)	64.1 (453)	
			3	18.2(28)	21.9 (155)	
			4	1.3 (2)	1.0 (7)	
			Fail	0 (0)	0 (0)	
	**5**	JP5:1 Elective (n = 861)	1	22.1 (34)	12.2 (86)	**< 0.001**
			2	54.5 (84)	47.7 (337)	
			3	20.1 (31)	31.5 (223)	
			4	3.2 (5)	8.6 (61)	
			Fail	0 (0)	0 (0)	
		JP5:2 Paramedical Elective (n = 861)	1	29.9 (45)	21.6 (153)	0.220
			2	50.6 (78)	56.0 (396)	
			3	17.5 (27)	20.2 (143)	
			4	2.6 (4)	2.1 (15)	
			Fail	0 (0)	0 (0)	

^1 ^Mark bands were categorised into their 5 locally-used bands: 1 = outstanding, CAS marks 18–20; 2 = very good, CAS marks 15–17; 3 = good, CAS marks 12–14; 4 = pass, CAS marks 9–11; Fail = CAS marks 0–8.

^2 ^P-value from chi-square test

Table 3 illustrates that there were significant differences between IC and non-IC students on several written exams in Years 1–3. Doing an intercalated degree was significantly associated with performance on the following written assessments taken before the intercalating year: Basic Science for Medicine (WR1:2, p < 0.001); Systems II (WR1:3, p = 0.009); Principles of Medicine III (WR2:4, p < 0.001) and Principles of Medicine IVb (WR3:2, p < 0.001). As shown in Table 4, after the IC year, IC students performed better than non-IC students on the only written exam in 4^th ^and 5^th ^year, the 4^th ^year written final (Specialist Clinical Practice II, WR4, p < 0.001).

In the 3^rd ^year OSCE (OSCE3), which takes place just before the IC year, IC students performed significantly better than their non-IC colleagues (p = 0.003). No significant associations were found for the 4^th ^or 5^th ^year OSCEs (OSEC4 p = 0.015 and OSCE5 p = 0.025) although the general trends were in the same direction.

A significant association between student performance and intercalating in the 3^rd ^year Community Course III (JP3, p = 0.003) and 5^th ^year Elective project (JP5:1, p < 0.001) was also seen.

Table 5 shows, that after adjustment for cohort, maturity, gender and baseline (3^rd ^year) performance of the matching exam type, doing an IC degree was significantly associated with attaining CAS marks of 18–20 in several of the 4^th ^and 5^th ^year exams. Students with an IC degree were over five times more likely than non-IC students to attain CAS marks of 18–20 in the 4^th ^year written Specialist Clinical Practice exam (WR4, adjusted odds ratio 5.17, 95% confidence interval 2.03–13.13). Similarly, IC students were almost three times more likely than non-IC students to attain CAS marks of 18–20 in the 4^th ^year OSCE (OSCE4) and they were more than five times more likely than non-IC students to attain CAS marks of 18–20 in the 5^th ^year Elective Project (JP5:1).

**Table 5:** Multivariate Analysis of Intercalated Student Performance versus non-intercalated student's performance for different assessment types (all models were based on 861 students)

**Assessment Type**	**Exam** **(Year)**	**Mark Band**^1^	**Unadjusted odds ratio (95% CI)**	**Adjusted**^2^**odds ratio (95% CI)**	**P-value**	**Nagelkerke R^2 ^for adjusted model**
Written	WR4 (4)	1	**5.95** **(2.71–13.05)**	**5.17** **(2.03–13.13)**	**< 0.001**	0.394
		2	**2.19** **(1.17–4.10)**	1.69 (0.85–3.38)	0.137	
OSCE	OSCE4 (4)	1	**2.97** **(1.39–6.34)**	**2.91** **(1.28–6.62)**	**0.001**	0.166
		2	1.69 (0.94–3.06)	1.48 (0.80–2.74)	0.217	
	OSCE5 (5)	1	**2.41** **(1.12–5.17)**	2.09 (0.92–4.75)	0.079	0.164
		2	1.17 (0.60–2.29)	1.07 (0.53–2.16)	0.844	
Essay	JP4 (4)	1	1.03 (0.20–5.24)	1.04 (0.19–5.76)	0.966	0.050
		2	0.72 (0.15–3.51)	0.70 (0.13–3.68)	0.972	
	JP5:1 (5)	1	**4.82** **(1.78–13.04)**	**5.13** **(1.85–14.23)**	**0.010**	0.057
		2	2.51 (0.99–6.37)	2.40 (0.93–6.20)	0.071	
	JP5:2 (5)	1	1.10 (0.35–3.49)	0.78 (0.23–2.66)	0.689	0.068
		2	0.73 (0.24–2.25)	0.53 (0.16–1.74)	0.294	

^1 ^Mark bands were CAS marks 0–11 (base group); 1 = CAS marks 12–17; 2 = CAS marks 18–20

^2 ^Adjusted for maturity, gender, cohort and baseline performance in the 3^rd ^year of the matching exam type

## Discussion

This study aimed to identify if students on a modern degree programme who did an optional intercalated degree performed better in subsequent undergraduate degree exams than their peers who did not intercalate. When we adjusted for performance in the early years of the course along with maturity and other covariates, intercalating was associated with gaining high CAS marks in three of the six degree assessments which took place after rejoining the course. These assessments reflected the range of assessment methods: written, clinical and essay-based.

We know that intercalating confers benefits in terms of gaining additional points in the academic ranking system for applications for FY1 posts. Our data indicates that intercalating was also associated with additional benefits in terms of improved performance in Years 4 and 5 of the MBChB. Improved performance will further contribute to higher academic ranking.

The relatively low numbers of students who choose to intercalate in Aberdeen (just under 18%) suggests that our students are not aware of these benefits – intercalating may become more attractive if they become so. Moreover, the proportion of Aberdeen students intercalating is well under the average proportion intercalating across all UK medical schools of 36% [2]. Thus, we believe we have scope to increase the number of Aberdeen students intercalating without any dilutational effect on the benefits [2]. However, it would be interesting to repeat this study for quality assurance purposes if we were to have a large increase in the numbers of students intercalating.

Intercalating students do significantly better in their final year elective project. This is a research project so it is not surprisingly that IC students, who had received more training and practice in the skills required to produce this kind of work than those who did not intercalate [7], do better than their colleagues.

Why do students who intercalate do better once they rejoin the medical course? We suggest this is probably due to a combination of maturity [20] and gaining new learning skills [2]. However, given the retrospective nature of this study, we were unable to directly measure if student learning styles changed as a result of intercalating, nor could we formally assess cause and effect.

Given that entry into the Aberdeen intercalated degree programme is based on attainment in third year, we expected IC students to do better than their colleagues on early exams. This was indeed apparent on several of the written exams. We propose that this might suggest that those who chose to do an intercalated degree are more academically-inclined than the average student. Thus, rather than an intercalated degree encouraging entry into academic medicine [4-6], it may be that those students who are more academic are attracted to intercalating as the first step of an academic career [8]. Further research is required to explore this possibility. Nonetheless, we did adjust the multivariate analysis for performance in 3^rd ^year of the matching exam type and therefore our overall findings are not simply a reflection of a higher academic ability of the IC students.

Intercalating students did better than their colleagues on the 3^rd ^year clinical (OSCE) exam. This pattern was maintained when the IC students rejoin the MBChB programme. IC students have no clinical contact in their intercalating year, so any improvement is likely due to maturity [20] and/or better learning strategies [2].

Younger students were more likely to do an IC degree. This may be associated with the fact that many older students were graduates, or simply with the fact that more mature students may have financial and/or family commitments, beyond those of the average school-leaver, which make an additional year of study difficult. While one would not expect a graduate to do an IC degree, we were initially surprised that no overseas student had ever intercalated. The most probable explanation for this is additional cost, especially in light of the increased amount overseas students pay in fees.

This is the first study exploring the short-term benefits, in terms of improved performance on subsequent undergraduate assessment, of intercalating in a population of students doing a modern (post Tomorrow's Doctors [13]) MBChB in which intercalating is optional (as is the case for the majority of UK medical schools). As far as we are aware, there is no published comparison data available on the breakdown of students who intercalate at other institutions. This paper presents retrospective, observational data derived from a single institution, so we would not claim that our results are necessarily applicable to other medical schools. However, we were able to look at five cohorts of students, giving the study high numbers and statistical strength in terms of power. We also looked at data from all students intercalating, not only those doing one intercalated option [11].

The design of this study does not allow for exploration of whether intercalating predisposes a medical student towards an academic career, or whether those who are predisposed towards an academic career are more likely to intercalate. If the former, the short-term gains from intercalating identified in this study may attract more medical students to intercalate, and hence, more graduates to academic medicine.

## Conclusion

In conclusion, intercalating has been reported to be beneficial to learning to learn and to an academic medicine career. Having an Honours degree contributes to academic ranking for Foundation Year applications in the UK. This study adds that intercalating was associated with improved performance in degree assessments after students rejoined the MBChB. Improved performance was measurable on a range of assessment methods. It may be that the short-term gains from intercalating identified in this study may attract more medical students to intercalate. We suggest this study be replicated in other UK medical schools to explore whether the short-term benefits associated with an intercalated degree that we identified at Aberdeen, are widespread. Long-term follow-up is required to identify if doing an optional intercalated degree as part of a modern medical degree is predictive of following a career in academic medicine.

## Competing interests

The authors declare that they have no competing interests.

## Authors' contributions

HS and JC had the original idea for this study. All authors were involved in the design and execution of the project. AM and AJL carried out the statistical analysis. JC wrote the first draft of the paper. All authors read and approved the final manuscript.

## Pre-publication history

The pre-publication history for this paper can be accessed here:


